# Viral replication organelles: the highly complex and programmed replication machinery

**DOI:** 10.3389/fmicb.2024.1450060

**Published:** 2024-07-31

**Authors:** Hao Deng, Hongwei Cao, Yanjin Wang, Jiaqi Li, Jingwen Dai, Lian-Feng Li, Hua-Ji Qiu, Su Li

**Affiliations:** State Key Laboratory for Animal Disease Control and Prevention, National African Swine Fever Para-reference Laboratory, National High Containment Facilities for Animal Diseases Control and Prevention, Harbin Veterinary Research Institute, Chinese Academy of Agricultural Sciences, Harbin, China

**Keywords:** viral replication organelles, viral helicase, DNA sliding clamp, viral DNA ligase, viral polymerase, antiviral drugs

## Abstract

Viral infections usually induce the rearrangement of cellular cytoskeletal proteins and organelle membrane structures, thus creating independent compartments [termed replication organelles (ROs)] to facilitate viral genome replication. Within the ROs, viral replicases, including polymerases, helicases, and ligases, play functional roles during viral replication. These viral replicases are pivotal in the virus life cycle, and numerous studies have demonstrated that the viral replicases could be the potential targets for drugs development. Here, we summarize primarily the key replicases within viral ROs and emphasize the advancements of antiviral drugs targeting crucial viral replicases, providing novel insights into the future development of antiviral strategies.

## 1 Introduction

Viral infections usually lead to the changes in intracellular cytoskeletal proteins, lipid synthesis and transport, membrane structures, and the endoplasmic reticulum (ER) (Schmid et al., 2014; Belov, 2016; Morita and Suzuki, 2021; Lan et al., 2023), many of which are hijacked by viruses for the formation of viral replication organelles (ROs). Similar to the inclusion bodies, ROs are closely related to viral replication and immunoevasion (Wu et al., 2022). More importantly, ROs provide an optimal replication environment for the viral replicases to enhance viral replication efficiency.

Compared with the small DNA viruses with limited genome coding capacity, the nucleocytoplasmic large DNA viruses, such as herpes simplex virus (HSV), initiate a programmed replication model by binding viral replicases to DNA (Packard and Dembowski, 2021). Among these, the functions of key replicases, such as the helicase UL5, sliding clamp UL42, and polymerase UL30 are precisely regulated to ensure the HSV replication (Zarrouk et al., 2017; Bermek and Williams, 2021; Cohan and Frappier, 2021). Additionally, the monkeypox virus (MPXV) helicase D5 and the African swine fever virus (ASFV) helicase C962R both form complex multimeric structures (Shao et al., 2023a; Li et al., 2024). Moreover, MPXV forms a specific forward sliding clamp, and the ASFV sliding clamp pE301R exists in two oligomeric states (Li et al., 2023; Peng et al., 2023a; Shao et al., 2023b; Wu et al., 2023b), which ensures the efficient and accurate replication of MPXV and ASFV. Research on RNA viral replicases have focused on RNA-dependent RNA polymerase (RdRp), a crucial polymerase involved in RNA virus replication that contains a highly conserved catalytic domain (Lu et al., 2021). Additionally, the molecular mechanisms of synthesis initiation and the nucleotide addition cycle (NAC) catalyzed by RdRps in various RNA viruses have been elucidated (Gong and Peersen, 2010; Shu and Gong, 2016). In primer-dependent synthesis, severe acute respiratory syndrome coronavirus 2 (SARS-CoV-2) nsp8 acts as a primase and, together with accessory factors nsp7 and RdRp nsp12, forms the minimal replicase complex to initiate genome synthesis (Gao et al., 2020). Concurrently, *de novo* synthesis is prevalent in the initiation of RNA virus genome synthesis. The formation of the bent conformation at the 3′ end of the Ebola virus (EBOV) RNA is crucial for RdRp template recognition (Peng et al., 2023b). Influenza virus (IAV) exhibit two mechanisms of synthesis initiation (Te Velthuis and Oymans, 2018; Fan et al., 2019). Furthermore, the NAC is essential for the elongation phase of RNA virus replication. Analysis of the six-state model of the poliovirus (PV) RdRp 3Dpol NAC and the seven-state model of the enterovirus 71 (EV71) RdRp 3Dpol NAC has provided a deeper understanding of the complex mechanisms of RNA virus NAC (Gong and Peersen, 2010; Shu and Gong, 2016). These studies gradually fill the gaps in fundamental theories related to viral replication and provide novel clues for the development of antiviral drugs. DNA virus polymerase inhibitors, such as acyclovir (ACV) and helicase-primase inhibitors, such as amenamevir, as well as RNA virus RdRp inhibitors, such as remdesivir, have been used to combat viral infections (Agostini et al., 2018; Kausar et al., 2021; Sato et al., 2021).

Currently, some viral replicases and antiviral drugs targeting the replicases have been identified. However, numerous challenges still exist. The precise structures and functions of replicases or the composition of replication complexes remain largely unknown. In addition, the mutations in viral replicases result in the drug resistance. Addressing these challenges will provide novel insights into the intricate regulatory mechanisms underlying viral genome replication. These findings may also pave the way for the development on novel antiviral drugs. Therefore, this review is focused on the research progress of viral replicases and the development of antiviral drugs targeting viral replicases. Additionally, we also discuss the current research limitations and future directions, aiming to provide relevant insights for the study of viral replication mechanisms and the development of therapeutic strategies for related diseases.

## 2 Composition of the viral ROs

**Figure 1:**
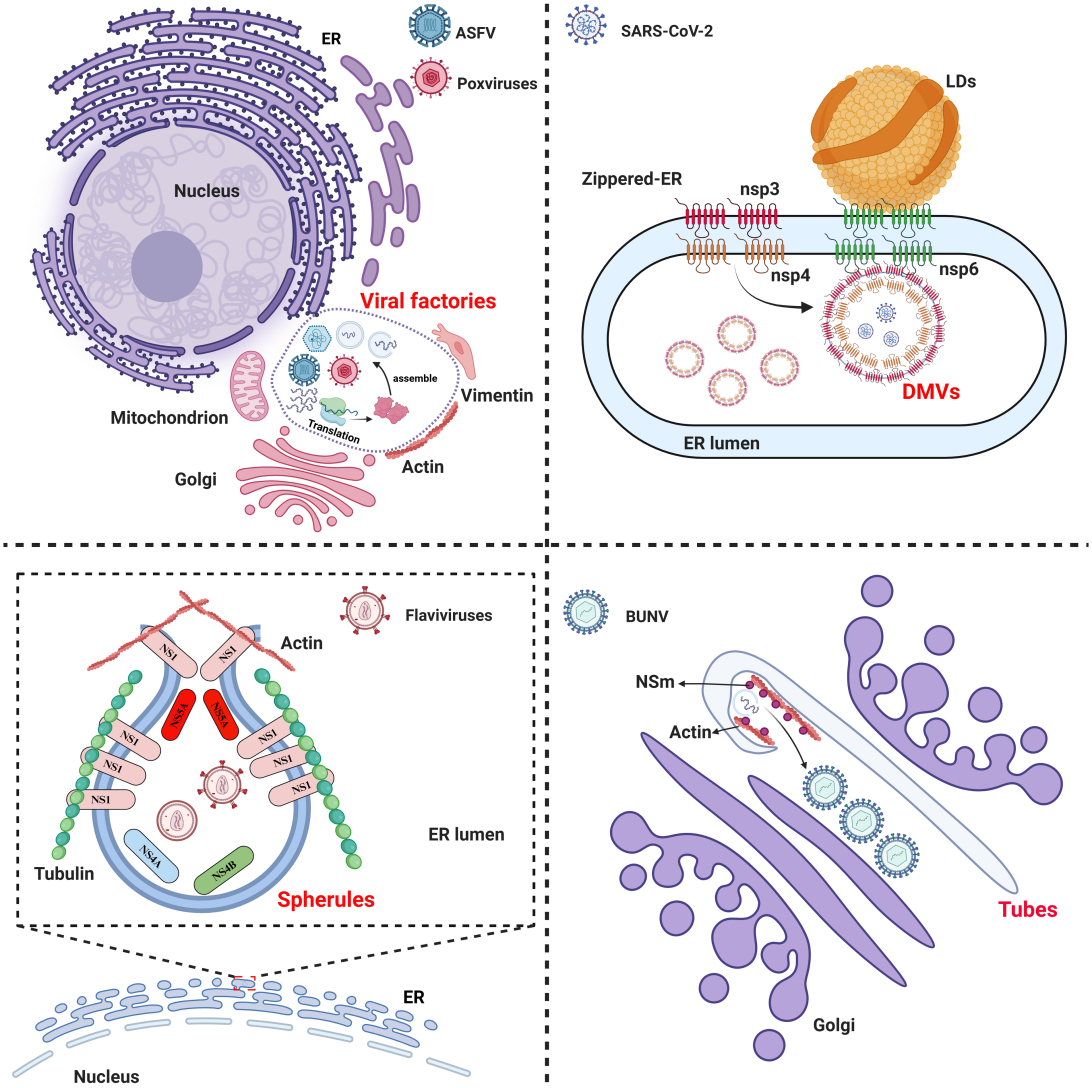
Schematic diagram of the components of viral replication organelles (ROs) in the cytoplasm. Viral factories: represented by African swine fever virus (ASFV) and poxviruses, the ROs are formed by altering the morphology and positioning of the cellular membrane systems and cytoskeletal proteins to generate larger ROs. Spherules: generated by flaviviruses, the invaginated vesicle is formed by viral proteins that remodel the ER lumen and interact with cytoskeletal proteins. Double-membrane vesicles (DMVs): induced by SARS-CoV-2, the ROs are formed by the viral nonstructural proteins nsp3/4, which create a zippered-ER that invaginates to produce a double-membrane structure. Additionally, there DMVs form complex with nsp6 and lipid droplet (LDs) and LDs provides the energy needed for DMVs development. Tubes: created by Bunyamwera virus (BUNV), this tubular membrane structure is formed by the viral NSm protein and actin in the Golgi apparatus.

Viral infection remodels the cellular endomembrane systems and cytoskeleton to form viral ROs (Wu et al., 2022), revealing a crucial step in the virus life cycle. The viral ROs formed in the cytoplasm can be categorized into four main types: (1) viral factories, represented by ASFV and poxviruses; (2) spherules, generated by flaviviruses; (3) double-membrane vesicles (DMVs), induced by coronaviruses; and (4) tubes, created by Bunyamwera virus (Figure 1). In addition to the cytoplasm, DNA viruses can also form ROs in the nucleus, as observed in HSV (Zhang et al., 2024).

ASFV and poxviral infections induce the rearrangement of cytoskeletal proteins, such as actin and vimentin, along with organelles including mitochondria, Golgi apparatus, and the ER within the cytoplasm. These components are relocated to the perinuclear area to form viral factories (Schmid et al., 2014). Flaviviruses and classical swine fever virus (CSFV) hijack and remodel the ER by using the nonstructural proteins (NS1, NS4A/4B, and NS5A) to form spherules, which benefit the viral replication (Cheng et al., 2023; Ci et al., 2023). In addition to organelles, the flaviviral NS1 protein orchestrates the recruitment of cytoskeletal proteins to form spherules through its interaction with the nuclear membrane proteins SUN2 and nesprin-1/-2 (Huang et al., 2024). SARS-CoV-2 forms DMVs at the ER through its viral proteins nsp3/4 (Pahmeier et al., 2023). Furthermore, the nsp6 protein of SARS-CoV-2 homodimerizes to create a zipper-like ER that connects with the DMVs and recruits lipid droplets (LDs), thereby providing essential fatty acids for DMV development (Ricciardi et al., 2022). Bunyamwera virus (BUNV) utilizes its NSm protein and actin as scaffolds, and assembles into tubular structures through interactions with Golgi membranes (Fontana et al., 2008). Interestingly, HSV, which replicates in the nucleus, orchestrates the formation of ROs through the nuclear molecular rearrangement without involving cytoplasmic membrane structures (Chang et al., 2011).

Viruses usually hijack the cellular endomembrane systems to establish a specialized microenvironment for viral replication and immune evasion (Zhang et al., 2024). Cytoskeletal proteins provide structural support for intracellular and intercellular viral spread through the interactions with viral proteins to facilitate viral replication. Additionally, cytoskeletal proteins are involved in endomembrane systems remodeling and the formation of the ROs during viral infection (Zhang et al., 2024). However, the involvement of organelles and cytoskeletal proteins in the formation of the ROs remains largely unknown, and the ROs are a hot topic in the field of virology research.

It is well accepted that the formation of viral ROs is crucial for viral replication. The exploitation of the cellular endomembrane systems and cytoskeletal proteins by viruses for the formation of the ROs is the initial stage of the virus life cycle. More specifically, the functional role of ROs in viral replication is to concentrate viral replicases, such as helicases, polymerases, ligases, and cofactors, such as sliding clamp proteins, thereby facilitating viral replication.

## 3 Critical viral replicases involved in viral genome replication

### 3.1 Viral DNA helicases: essential replication initiation components of DNA viruses

During DNA virus replication, the unwinding of the DNA double helix is a prerequisite for subsequent genome replication, with DNA helicases playing an essential role in this process (Weller and Kuchta, 2013). Several DNA viral helicases have been identified, including the ASFV pC962R, MPXV D5, bovine papillomavirus (BPV) E1, and simian virus 40 (SV40) large tumor antigen, which belong to the AAA+ proteins (ATPases associated with diverse cellular activities) in the SF3 family (Gai et al., 2004; Joo et al., 2019; Shao et al., 2023a; Li et al., 2024), indicating the conservation of the helicases of various DNA viruses.

**Figure 2:**
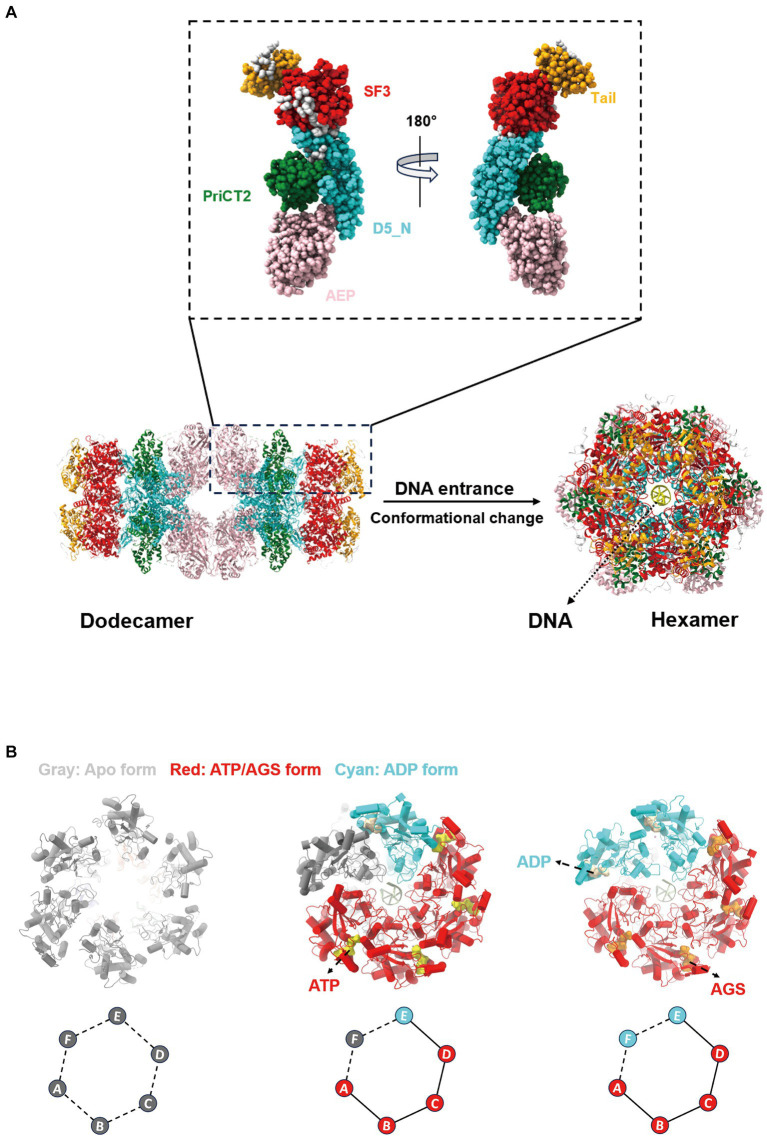
Changes in the conformation of the African swine fever virus (ASFV) pC962R and the monkeypox virus (MPXV) D5 SF3. **(A)** Structural diagram illustrating the conformational change of the helicase pC962R from a dodecameric form (Protein Data Bank [PDB] ID: 8IQH) to a hexameric state (PDB ID: 8IQI), with five different colors representing the individual domains of pC962R. **(B)** Structural transitions of the helicase D5 SF3 in the Apo form (PDB ID: 8HWC), ATP-ADP-Apo-ssDNA form (PDB ID: 8HWA), and AGS-ADP-ssDNA form (PDB ID: 8HWG). Gray, red, and cyan represent different states of the D5 SF3. The colors and letters at the vertices of the diamond-shaped diagrams correspond to the schematic representations denoting distinct states of the D5 protein SF3. The solid lines indicate tight connections and the dashed lines indicate loose connections. AGS, ATP-gamma-S (a nonhydrolyzable ATP analog). The models were generated using the ChimeraX software downloaded from https://www.cgl.ucsf.edu/chimerax/.

**Figure 3:**
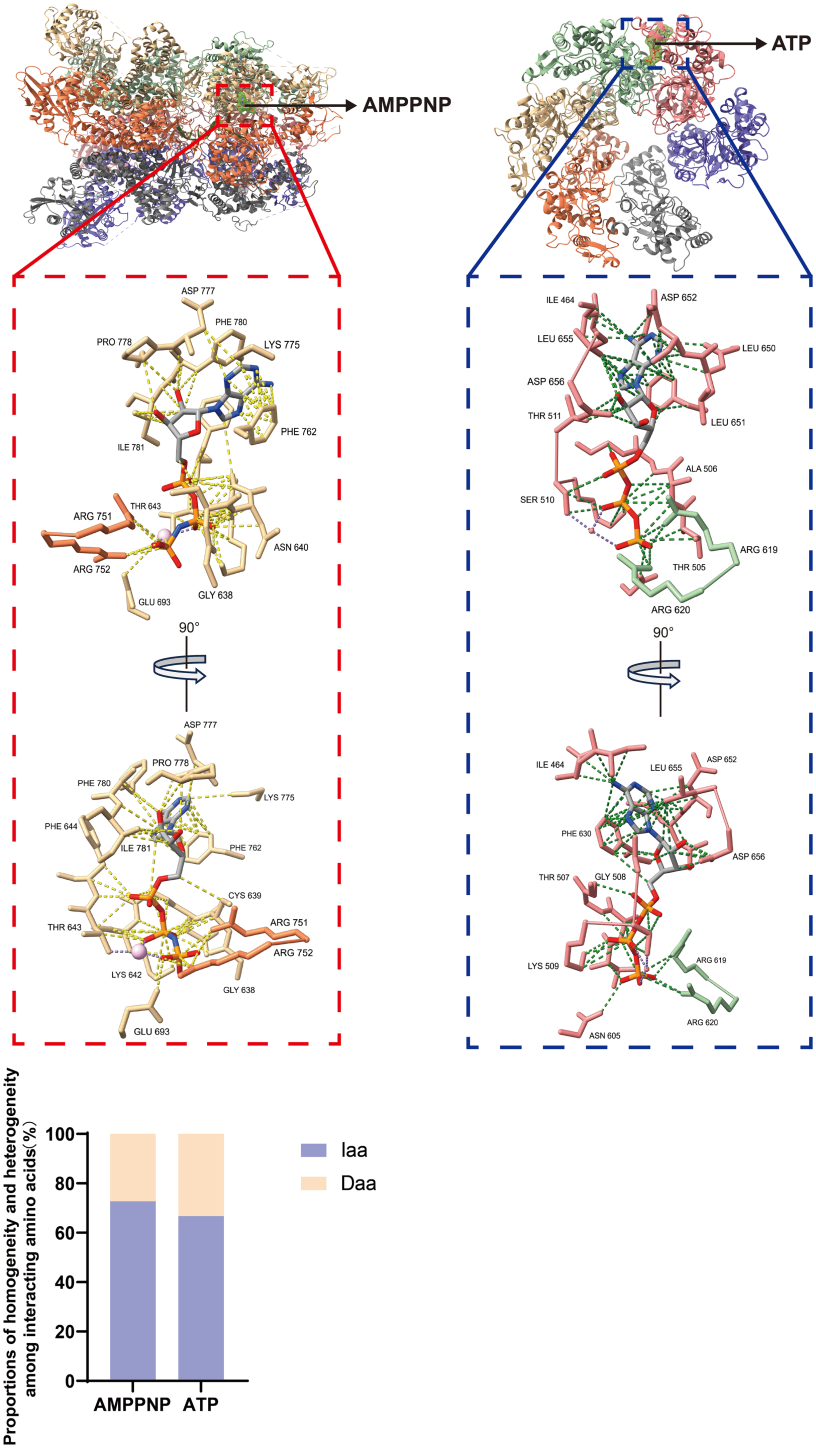
Interactions between ligands and adjacent amino acids (aa) in the SF3 of the pC962R or D5. Atomic models of the interaction between AMPPNP in the pC962R SF3 and ATP in the D5 SF3 with adjacent aa. The models were generated using the ChimeraX software downloaded from https://www.cgl.ucsf.edu/chimerax/. Proportions of the pC962R SF3 and D5 SF3 aa are involved in the interactions with AMPPNP and ATP, respectively. AMPPNP, adenylyl-imidodiphosphate (a nonhydrolyzable ATP analog); Iaa, identical amino acids; Daa, different amino acids. The bar chart was generated using the Prismchs software.

The ASFV pC962R can transit from a dodecameric form to a hexameric state through interaction with DNA. SF3 is assisted by the PriCT2 and D5_N domains, along with the C-terminal tail domain, and, under ATP catalysis, exerting DNA 3′-5′ helicase activity (Shao et al., 2023a) (Figure 2A). Similarly, the C-terminal SF3 helicase activity of the MPXV D5 is modulated by both the N-terminal primase domain and ATP. The N-terminal primase domain inhibits the C-terminal helicase activity through conformational changes, while disruption of the interaction between the primase and helicase significantly enhances the helicase function of the D5 (Li et al., 2024). Therefore, further study is required to elucidate the molecular mechanism by which the MPXV helicase D5 unwinds, particularly regarding the regulation of primase domain conformational changes by viral or host factors, thereby impairing the unwinding efficiency of D5. Additionally, ATP hydrolysis is a crucial requirement for the functional activity of the SF3 domain (James et al., 2003). A recent study revealed that the ATP hydrolysis cycle regulates the degree of tight binding between the hexameric SF3 domains of MPXV D5 (Li et al., 2024) (Figure 2B). The profound biological significance of SF3 domains in promoting MPXV DNA replication warrants further investigation. Furthermore, an analysis of the amino acids (aa) involved in the interaction between the SF3 domains of the ASFV pC962R or the MPXV D5 and their ligands (ATP or AMPPNP) revealed aa similarities in the binding of the SF3 domains and ligands (Figure 3) (Shao et al., 2023a; Li et al., 2024). Thus, investigation of the analogous aa binding characteristics of the ATP-driven DNA virus helicases may facilitate the development of broad-spectrum antiviral drugs targeting the ATP-binding pockets.

Other DNA viral helicases can form heterologous oligomers with other viral proteins. For instance, the HSV helicase UL5 forms heterotrimers with the primase UL52 and the noncatalytic subunit UL8 (Bermek and Williams, 2021). However, UL5 is known to possess seven highly conserved SF1 helicase family motifs. Mutations in motifs I and IV inhibit helicase activity without affecting the interaction between UL5 and UL52 or UL8 (Bermek and Williams, 2021). Therefore, it is worth investigating whether disruption of the UL5-UL52-UL8 complex suppresses the helicase activity of UL5 and other biological functions of the complex. Addressing this question will enhance our understanding of the regulatory mechanism of the human cytomegalovirus (HCMV) helicase (UL105)-primase (UL70)-accessory factor (UL102) complex (Ligat et al., 2018), as well as other analogous DNA viral helicase complexes. A comprehensive investigation of the helicase functions will contribute to a better understanding of the biological characteristics of DNA viral replication.

### 3.2 Sliding clamp proteins: the critical processivity factors in nucleocytoplasmic large DNA viruses

**Figure 4:**
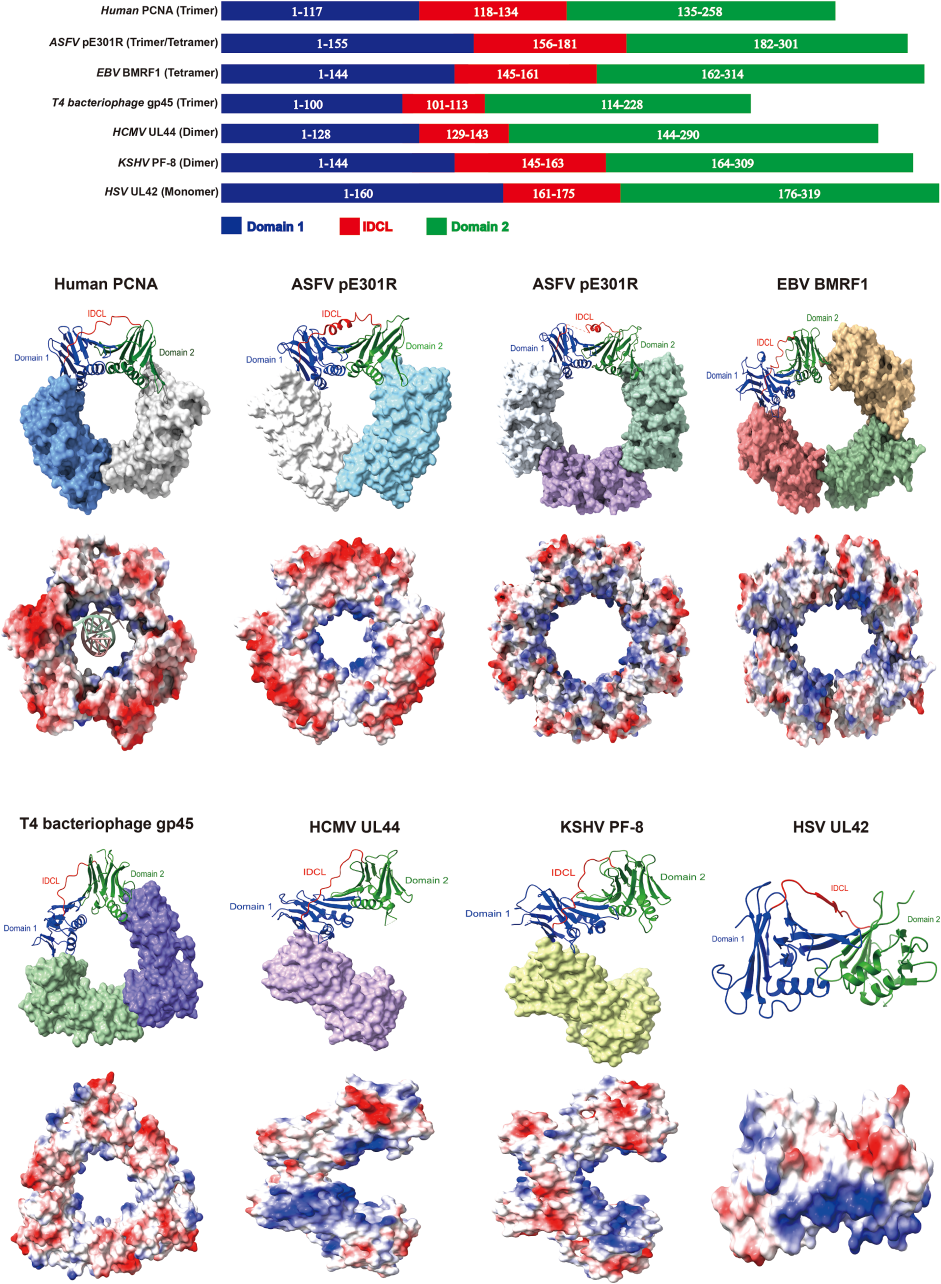
The sliding clamp proteins of DNA viruses exhibit comparable domain architectures. Domain architectures, oligomeric states and electrostatic potential surface distributions of human proliferating cell nuclear antigen (PCNA) and sliding clamps from DNA viruses. Red indicates negative and blue represents positive in the lower panel of the sphere style of the human PCNA and sliding clamps. PDB ID: 6GIS (human PCNA), 8I6G and 8ITE (ASFV pE301R), 2Z0L (EBV BMRF1), 1CZD (T4 bacteriophage gp45), 1T6L (HCMV UL44), 3HSL (KSHV PF-8), and 1DML (HSV UL42). The models were generated using the ChimeraX software downloaded from https://www.cgl.ucsf.edu/chimerax/.

The sliding clamp proteins of DNA viruses exhibit remarkable structural and functional similarities with the human proliferating cell nuclear antigen (PCNA) (Li et al., 2023). The human PCNA forms homotrimers, with each monomer consisting of two folded similar domains connected by an interdomain-connecting loop (IDCL). Domain 1 spans the residues 1 to 117, domain 2 spans the residues 135 to 258, and the IDCL spans the residues 118 to 134 (Gonzalez-Magana and Blanco, 2020). These six domains assemble into a ring-shaped structure, with an outer layer comprising six *β*-sheets and an inner layer comprising 12 *α*-helices arranged around the central pore of the ring. The central pore is surrounded by positively charged aa, which can engage in electrostatic interactions with double-stranded DNA, serving as a processivity factor for DNA polymerases to facilitate genome replication (Gonzalez-Magana and Blanco, 2020). Considering the powerful function of PCNA, researchers have identified several sliding clamps of nucleocytoplasmic large DNA viruses, including ASFV (Li et al., 2023), Epstein–Barr virus (EBV) (Nakayama et al., 2010), T4 bacteriophage (Moarefi et al., 2000), HCMV (Appleton et al., 2004), Kaposi’s sarcoma–associated herpesvirus (KSHV) (Baltz et al., 2009), and HSV (Zuccola et al., 2000), which harbor the genes encoding oligomeric or monomeric DNA sliding clamp proteins, and exhibit structural, functional, and charge distributional similarities to that of human PCNA (Figure 4).

The ASFV pE301R and EBV BMRF1 proteins form homologous tetrameric sliding clamps through head-to-tail and head-to-head/tail-to-tail interactions, respectively (Nakayama et al., 2010; Li et al., 2023). However, others also indicate the existence of pE301R in the trimeric form (Shao et al., 2023b; Wu et al., 2023b), and further investigation is required to elucidate how ASFV precisely assembles multiple pE301R oligomers and whether different assembly states play distinct roles in the ASFV life cycle. Importantly, the mutations in C206 (involved in tail-to-tail interface formation) and R256 (responsible for DNA binding) of BMRF1 disrupt of the formation of sliding clamps, severely impairing the EBV replication (Nakayama et al., 2010), suggesting that the critical regions of the viral sliding clamp protein are essential for maintaining its function, serving as the potential targets for antiviral drugs. Furthermore, EBV hijacks the cellular PCNA for replication during the latent infection; however, EBV switches to utilizing the virus-encoded sliding clamp protein BMRF1 for replication during the lytic infection (Nakayama et al., 2010). Notably, when EBV employs the viral replication machinery, its genome replication efficiency is 100–1,000 times greater than that of the utilization of the cellular replication machinery (Nakayama et al., 2010). The replication mechanism represents a protective strategy for EBV during different stages of infection and serves as a reference for investigating the latent reactivation of other DNA viruses. Interestingly, the trimeric sliding clamp formed by the T4 bacteriophage gp45 protein exhibits lower stability in DNA binding than do the human PCNA and the *Escherichia coli* DNA sliding clamp *β*. The interaction between the gp45 protein and the polymerase is required for the binding of the gp45 protein to template DNA. However, among these, the most stable sliding clamp, the *β*-clamp, forms a dimeric ring structure (Kong et al., 1992; Yao et al., 1996; Moarefi et al., 2000). The difference in DNA-binding stability among sliding clamps from different organisms may be related to distinct replication mechanisms. Apart from conventional ring-shaped oligomers, DNA viral sliding clamp proteins, such as the HCMV UL44 and KSHV PF-8, form the C-type homologous dimers through head-to-head interactions, while the HSV UL42 sliding clamp protein exists as a monomer (Zuccola et al., 2000; Appleton et al., 2004; Baltz et al., 2009). Therefore, the comparative analyses of various sliding clamp proteins of DNA viruses may exhibit both common characteristics and differences in their structure and functions, providing novel targets for antiviral strategies.

Recently, researchers have revealed that the uracil-DNA glycosylase E4 protein encoded by MPXV interacts with the exonuclease domain (Exo) and N-terminal domain (NTD) of the F8 polymerase, and with the engagement of the A22 protein, the complex of A22, E4 and F8 forms a forward sliding clamp for viral genome replication (Peng et al., 2023a), diverging from the conventional backward sliding clamp. The distinctive sliding clamp protein exhibits structural similarities with the A20/D4 protein encoded by vaccinia virus (VACV) (Tarbouriech et al., 2017; Peng et al., 2023a), which enriches the current understanding of DNA sliding clamps. Although biochemical experiments have revealed the indispensable role of A20/D4 in VACV replication (Stanitsa et al., 2006), the VACV G8R protein was predicted to possess a sliding clamp protein structure, and treatment with PCNA-specific inhibitors results in the inhibition of viral replication (Da Silva and Upton, 2009). However, neither the structure nor the identification of the PCNA-like function of G8R by biochemical experiments was shown in the previous study. DNA sliding clamps play a pivotal role in the replication process of DNA viruses. The identification of sliding clamps encoded by DNA viruses could provide a significant theoretical foundation for the subsequent development of antiviral strategies.

### 3.3 Viral DNA polymerases: the “molecular motor” of the genome replication of DNA viruses

DNA viruses primarily depend on viral DNA polymerases to replicate viral genomes, a pivotal process in the virus life cycle (Packard and Dembowski, 2021). Despite the structural similarities between viral polymerases and cellular DNA polymerases, they also employ unique replication strategies. Notably, the viral DNA polymerases E9 (VACV), F8 (MPXV), UL30 (HSV), and UL54 (HCMV) all exhibit an open conformation characterized by palm, thumb, fingers, exonuclease, and distinctive N-terminal domains, which are typical features of B-family DNA polymerases (Tarbouriech et al., 2017; Zarrouk et al., 2017; Xu et al., 2023).

After the binding of primers to the template DNA, conformational changes in the thumb domain enhance the binding activity of the primer-template DNA. Furthermore, the finger domain of F8 rotates toward the palm domain, thereby facilitating the accommodation of deoxyribonucleoside triphosphates (dNTPs) within the groove formed by the finger and palm domains (Peng et al., 2023a). The interplay between the different domains of F8 and dNTPs facilitates the completion of the repetitive cycle of free dNTPs binding to the template strand (Peng et al., 2023a). The replication mechanism is similar to that of complementary strand synthesis catalyzed by the HSV UL30 or HCMV UL54 proteins (Zarrouk et al., 2017). However, the precise mechanism of the 5′-3′ polymerase activity of VACV E9 remains to be determined. Nonetheless, it catalyzes the annealing of single-stranded DNA, a function highly specific among the B-family DNA polymerases (Tarbouriech et al., 2017). Moreover, the E9 protein exhibits a stronger affinity to template DNA in low physiological salt buffers and displays sequence preferences in recognizing primer-template junctions (DeStefano et al., 2022). However, the biological significance of these preferences remains to be determined.

The diverse replication mechanisms of DNA viruses depend on exploitation of the cellular replication machinery. In addition to viral DNA polymerases, several DNA viruses hijack cellular DNA polymerases to facilitate replication. For instance, minute virus of mice (MVM), a single-stranded linear DNA virus, initiates replication by forming hairpin structures at palindrome sequences on the 5′ end of the genome, which is catalyzed by host DNA polymerase *δ* (Cossons et al., 1996). The hepatitis B virus (HBV) genome exhibits a distinctive characteristic: the transition of the relaxed circular DNA (rcDNA) into the covalently closed circular DNA (cccDNA) is required for replication (Tsukuda and Watashi, 2020). The process involves host DNA polymerases *α* and *κ* (Qi et al., 2016; Tang et al., 2019). The polymerase *α* participates in repairing gaps in the negative strand of rcDNA, leading to the generation of cccDNA (Tang et al., 2019); however, the biological functions of polymerase *κ* require further elucidation. The utilization of both viral and cellular DNA polymerases by DNA viruses confers a survival advantage, which represents a novel strategy in viral evolution, thereby facilitating viral replication.

### 3.4 Viral DNA ligases: the “adhesive” for single-strand gaps in DNA virus genomes

DNA ligases have been identified in various DNA viruses, with significant research efforts focused on the Chlorella virus DNA ligase (ChVLig), which comprises three main structural domains: the nucleotidyltransferase (NTase) domain, the OB-fold domain (OB), and the virus-specific *β*-hairpin latch module (Samai and Shuman, 2011b). The NTase domain induces conformational changes at the DNA nick site, the OB binds to the DNA minor groove at the nick position, and the *β*-hairpin latch module occupies the DNA major groove. The latter two domains interact with the NTase, thereby synergistically maintaining and stabilizing their respective functions (Samai and Shuman, 2011a,b). The functions of ChVLig primarily involve the following three processes. Initially, the ligase initiates its action by utilizing ATP, leading to the cleavage of ATP *α* phosphate group and the release of pyrophosphoric acid while simultaneously forming a covalent ligase-adenylate intermediate. Subsequently, AMP is transferred to the 5′-phosphate of the nick, thereby creating DNA adenylate. Finally, the 3′-OH group of the nick initiates an attack on DNA adenylate, facilitating the connection of polynucleotides and the release of AMP (Samai and Shuman, 2012). ChVLig can effectively catalyze both the ligation of DNA–RNA hybrids and DNA ligation, revealing its unique function in DNA repair and recombination (Lohman et al., 2014). Compared with that of ChVLig, the structure of the ASFV DNA ligase is distinctive, with its NTD being responsible for DNA binding and assisting in the formation of the ligation complex. The adenylation domain (AD), particularly Asn153 and Leu211, as well as the OB Leu402 and Gln403, are crucial for catalyzing chain ligation reactions (Chen et al., 2019), highlighting the diversity of DNA virus ligases. It is imperative to analyze the functions and structure of the active site of ASFV DNA ligase. Future research is essential to validate and pinpoint potential drug targets and assess the effectiveness and safety of candidate inhibitors by homology modeling and biochemical analysis. Intriguingly, the ligase-mutant VACV recruits host cell DNA ligase I to ROs to compensate for their own DNA ligase functions (Moss, 2013), indicating a robust viral adaptability of VACV. The findings shed light on how VACV employs host cell DNA ligase I to facilitate its replication, thereby enhancing our understanding of the interactions between VACV and the host cell.

In general, current studies are focused on the structures or functions of the DNA ligases encoded by other DNA viruses. A series of studies, represented by the ChVLig, are currently elucidating the important roles of DNA ligases in the field of virology. These findings have garnered widespread attention in the scientific community, revealing the important strategies by which viruses exploit DNA ligases. Additionally, these studies contribute to the understanding of newly emerging or re-emerging DNA virus ligases, providing novel insights for the precise targeting of antiviral drugs in the future.

### 3.5 The RdRps in RNA virus replication: a core component in the replicase complex

Research on RNA virus RdRp functions has focused primarily on the initiation and elongation phases, which are essential for comprehending the operational mode of RNA virus replication (Li et al., 2020). RNA viruses initiate RNA synthesis by primer-dependent or *de novo* synthesis mechanisms (Zhang et al., 2021a). In primer-dependent initiation, the replication process of the template chain of RNA viruses requires the participation of auxiliary factors, such as primer enzymes, which form replication complexes with RdRps (Wang Q. et al., 2020). Functional studies on the replication complex of coronaviruses have revealed that the nsp12 of SARS-CoV-1/2 serves as the viral RdRps. Upon combination with nsp7 and nsp8, the continuous synthesis of nsp12 can be enhanced by the replication complex nsp12-nsp7-nsp8 (Subissi et al., 2014; Gao et al., 2020; Peng et al., 2020). Furthermore, the G motif of RdRps in coronaviruses can recognize primer-template junction (Gorbalenya et al., 2002). The *de novo* synthesis mechanism is widely observed in several RNA viruses. It has been shown that the formation of the initiation complex (IC) is crucial for viral RNA *de novo* synthesis (Wu et al., 2023a). During the initiation of viral replication, the hepatitis C virus (HCV) NS5B protein undergoes a transition from an apoprotein (APO) form to an activated state. The *β* loop and *C*-terminus open the active site, allowing the RNA template and initiating nucleotide to form the IC for replication (Appleby et al., 2015). EBOV, one of the nonsegmented negative-stranded RNA viruses (nsNSVs), forms the IC with its L polymerase, the auxiliary factor VP35, and the RNA adopts a stable 3′ bent conformation to initiate the replication of the viral genome. Moreover, RNA 3′-specific conformational changes are critical for the replication initiation (Peng et al., 2023b), providing valuable insights into the initiation mechanism of nsNSVs. These findings are beneficial for understanding the initiation of replication mechanisms in nsNSVs. Compared with nsNSVs, the segmented negative-stranded RNA viruses (sNSVs) exhibit specific genome structures (Hamele et al., 2023). IAV exhibit two distinct initiation mechanisms during the replication of viral RNA (vRNA). The homodimers formed by the RNA polymerases (comprising PB1, PB2, and PA proteins) employ the generated loop for the pppA/G-dependent *de novo* initiation of vRNA and internal reassortment of complementary RNA (cRNA), which highly depends on the conformational transition of the IAV RdRp (Te Velthuis and Oymans, 2018; Fan et al., 2019). The underlying mechanisms for the coexistence of these two types of *de novo* replication initiation and practical significance for the regulation of the viral replication cycle require further in-depth investigation, which will provide a promising avenue for the development of innovative antiviral drugs targeting RdRps.

Additionally, the priming element (PE) in the thumb region of RdRps serves as a crucial component for the *de novo* synthesis of RNA. Structural variations in PE are observed among RNA viruses from different genera, such as *Hepacivirus*, *Pestivirus*, and *Flavivirus* (Zhang et al., 2021a). The diversity impedes the exploration of the mechanisms of *de novo* synthesis and the identification of conserved motifs of RNA. A previous study revealed the importance of the aa at position 671 and the C-terminal tail of the thumb subdomain PE of the CSFV NS5B protein in RNA *de novo* synthesis (Zhang et al., 2021a). During the initiation to elongation transition of the dengue virus serotype 2 (DENV2) RdRp, the conformational changes in PE contribute to stabilizing the RdRp elongation complex (EC) (Wu et al., 2023a). Therefore, the *de novo* synthesis of viral RdRps containing PE may involve a universal catalytic mechanism. Investigating the general conformational changes in the RdRps regulating the initiation stage of genome replication will provide directions for developing novel strategies to inhibit RNA virus replication.

**Figure 5:**
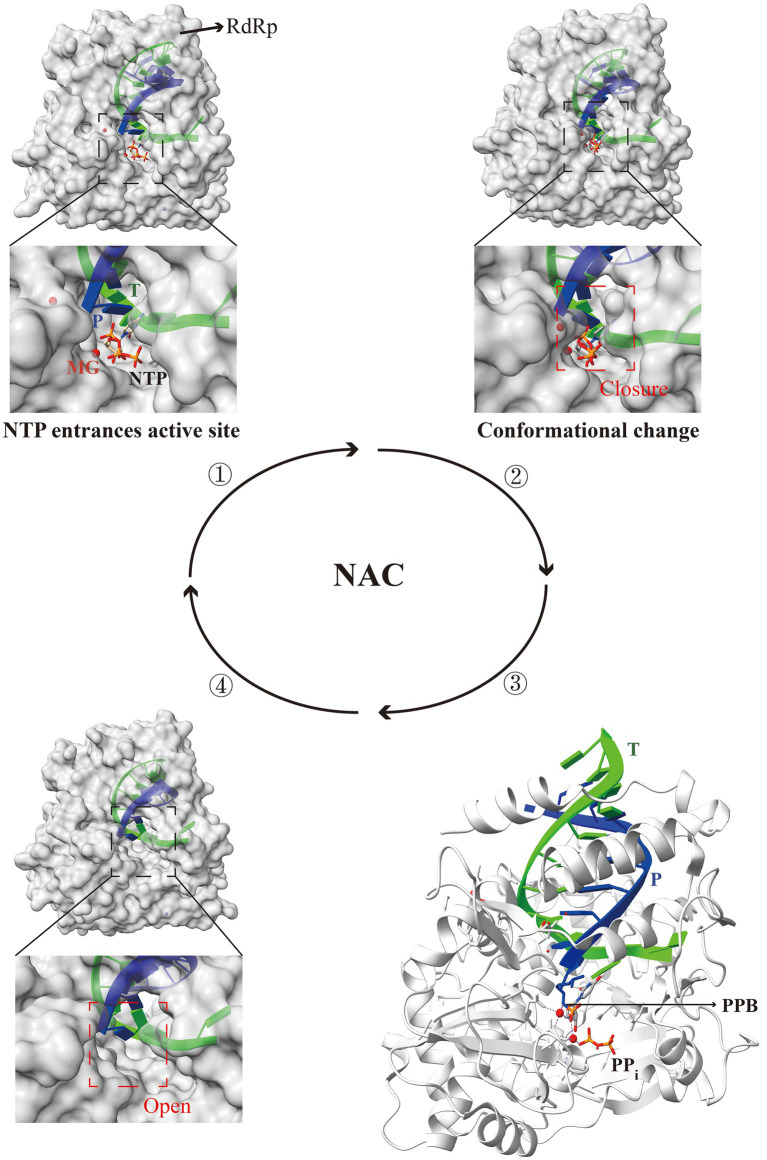
Structural patterns of nucleotide addition cycle (NAC) modulation by the RdRp active center in RNA viruses. The RdRp active center completes one NAC through four steps: entry of NTP into the active site, closure of the active site, formation of the phosphodiester bond, and reopening of the active site. Briefly, RdRp binds to the template strand with NTP at the active site, inducing closure and conformational changes that confer catalytic activity. Two Mg^2+^ ions enable NTP to form phosphodiester bonds with the new RNA chain. The absence of Mg^2+^ results in the destabilization of the active site, leading to the opening of the RdRp complex. The open active site facilitates the binding of new NTP, thereby repeating the cycle for the synthesis of progeny RNA. T, template (lime); P, product (blue); PPB, phosphodiester bond; PDB ID: 5F8I (one), 7W9S (two), 5F8M (three), and 6LSH (four). The models were generated using the ChimeraX software downloaded from https://www.cgl.ucsf.edu/chimerax/.

During the RNA virus genome elongation stage, the NAC is closely associated with RdRp (Gong, 2022). RdRp initially binds to the template strand with the complementary nucleoside triphosphates (NTPs) at the active site. Subsequently, closure of the active site occurs, and conformational changes triggered by the correct NTPs binding restrict access to the active site, resulting in a catalytically active state. In the catalytically active state, two Mg^2+^ ions are arranged around the newly synthesized RNA chain in a specific manner, allowing the substrate NTPs to undergo phosphotransfer and form phosphodiester bonds with the newly synthesized chain. In the absence of Mg^2+^ ions in the replication complex, the active site will no longer possess catalytic stability, and the RdRp complex will adopt an open conformation. Following the pairing of the template RNA with substrate NTPs via site transfer, RdRp with an open active site binds to new NTPs, thus repeating the cycle for the synthesis of progeny RNA (Shu and Gong, 2016; Wu and Gong, 2018; Wang et al., 2020b) (Figure 5). However, a NAC involves numerous chemical changes between RdRp and substrate NTPs, and deep understanding of the catalytic activities of RdRp holds significant importance.

Researchers have proposed an early six-state model for the PV RdRp 3Dpol NAC, indicating that the closure of the 3Dpol active site is associated with the replication template (Gong and Peersen, 2010). Further investigation into the catalysis and translocation mechanism of the EV71 3Dpol RdRp led to the construction of seven RdRp elongation complex conformations through three NACs, within which an asymmetrical relationship between the template-product RNA duplex in the translocation intermediate complex was identified (Shu and Gong, 2016). The unique interaction between the catalytic motif G of RdRp and the template strand “lock” is likely to the primary factor causing the asymmetric movement of the RNA double helix (Wang et al., 2020b). Recently, by constructing the conformation before the closure of the EV71 3Dpol active center, the K360 and R174 sites were shown to be associated with the catalytic efficiency of RdRp, and the subsequent examination revealed participation in nucleotide transfer (Li et al., 2022). However, whether these two catalytic key sites are conservation in other viral RdRps requires comparative analysis and validation using structural and biochemical experiments.

In light of this, the comprehensive investigation of the catalytic and regulatory mechanisms of RdRps in RNA virus replication is imperative for comprehending the distinctive characteristics of RNA viruses, facilitating the dissection of evolutionary characteristics and the development of antiviral strategies.

## 4 Targeting the key viral replicases for inhibitor development

Currently, a variety of antiviral drugs targeting replicases have been developed for DNA and RNA viruses, including HSV, HCV, YFV, IAV, coronaviruses, and the 2019 global pandemic SARS-CoV-2 (Table 1). The drugs generally exhibit broad-spectrum antiviral activity and promising applications in clinical trials. Among the drugs approved by the Food and Drug Administration (FDA), ACV, tenofovir, valganciclovir, and valacyclovir are viral DNA polymerase inhibitors (Kausar et al., 2021).

**Table 1:** Antiviral drugs that interfere with the normal functions of viral key replicases.

Drugs	Types	Targeted viral proteins	Viruses	Drug resistance	Drugs with similar targets and chemical structures	References
ACV	Nucleoside analog that mimics guanosine	DNA polymerase	HSV, VZV, EBV, CMV, and HHV-6	Yes	TDF, TAF, GCV, PCV, VACV, VGCV, BVDU, FCV, CDV, and FOS	Kausar et al. (2021), Majewska and Mlynarczyk-Bonikowska (2022), Schalkwijk et al. (2022), Wagstaff et al. (1994)
Amenamevir	Non-nucleoside analogs	DNA helicase-primase	HSV	Yes	PRT, BILS 179 BS, and T157602	Sato et al. (2021), Shiraki et al. (2021)
Remdesivir	Ribonucleotide analog	RdRp	EBOV, MERS-CoV, SARS-CoV, and SARS-CoV-2	Yes	RBV, GDV, SOF, FPV, and MOL	Agostini et al. (2018), Gordon et al. (2020a), Lu et al. (2021), Paladugu and Donato (2020), Tchesnokov et al. (2019), Wang et al. (2020a)
Nanobody	Single-chain antibody molecule	RdRp	IAV, PRRSV, CHIKV, and HCV	No	\	Deng et al. (2022), Fan et al. (2019), Thueng-in et al. (2012), Wang et al. (2019)

TDF, tenofovir disoproxil fumarate; TAF, tenofovir alafenamide; GCV, ganciclovir; PCV, penciclovir; VACV, valacyclovir; VGCV, valganciclovir; BVDU, brivudine; FCV, famciclovir; CDV, cidofovir; FOS, phosphonoformic acid; PRT, pritelivir; BILS 179 BS, 2-amino-thiazolylphenyl derivative; T157602, 2-amino thiazole compound; RBV, ribavirin; GDV, galidesivir; SOF, sofosbuvir; FPV, favipiravir; MOL, molnupiravir.

Valacyclovir, the prodrugs of ACV, can be metabolized in the body to form ACV after oral administration (Field and Vere Hodge, 2013). The bioavailability of valacyclovir is superior to that of ACV, and it also exhibits a longer half-life, enabling less frequent administration (Argenziano et al., 2023). ACV can block the extension and replication of viral DNA chains through the competitive inhibition of viral DNA polymerase, revealing the effective treatment of HSV infections (Schalkwijk et al., 2022). Additionally, ACV exhibits antiviral activity against varicella-zoster virus (VZV), EBV, cytomegalovirus (CMV), and human herpesvirus 6 (HHV-6) (Wagstaff et al., 1994), but its efficacy against EBV-related diseases is 10-fold greater for TDF and 35-fold greater for TAF than for ACV (Drosu et al., 2020). Moreover, due to the suboptimal therapeutic outcomes of ACV in patients infected with CMV, as well as the emergence of resistance to HSV and VZV, researchers are focused on the design of antiviral strategies for other herpesviral targets (Majewska and Mlynarczyk-Bonikowska, 2022), aiming to achieve robust treatment effects. Therefore, a drug named amenamevir, which targets helicase-primase, has been approved in Japan for inhibiting herpesviral infections (Shiraki et al., 2021). The drug can bind to the helicase-primase complex, thereby inhibiting the ATPase, helicase, and primase activities. Compared with other helicase-primase inhibitors, such as pritelivir, 2-amino-thiazolylphenyl derivatives, and 2-amino thiazole compounds, as well as DNA polymerase inhibitors, such as ACV, valacyclovir, and amenamevir, exhibited a lower median effect concentration (EC_50_) value (Shiraki et al., 2021). However, resistance mutations have been observed upon amenamevir administration. Compared with those of wild-type viruses, the pathogenicity of the HSV primase UL52 R367H or S364G mutant viruses was reduced, while the growth kinetics and pathogenicity of the helicase UL5 K356N mutant viruses remained unchanged (Sato et al., 2021; Shiraki et al., 2021). During the interplay between the virus and amenamevir, a gradual accumulation of mutations occurs, leading to the emergence of a new dominant virus strain, suggesting the necessity for updating antiviral drugs targeting DNA viruses. When necessary, the conserved regions of key proteins involved in DNA virus replication, such as the DNA sliding clamp, should be explored as potential targets for the development of novel antiviral drugs.

Remdesivir, also known as GS-5734, was initially designed for treating HCV infection as an adenosine nucleoside triphosphate analog. However, its antiviral activities *in vitro* and *in vivo* have evoked interest in its potential for treating other viral infections, such as EBOV (Warren et al., 2016), Middle East respiratory syndrome–related coronavirus (MERS-CoV) (Gordon et al., 2020a), SARS-CoV (Agostini et al., 2018), and SARS-CoV-2 (Paladugu and Donato, 2020; Wang et al., 2020a). From a structural biology perspective, the underlying mechanism by which remdesivir interferes with the functions of SARS-CoV-2 nsp12 has been elucidated. The drug undergoes hydrolysis to form remdesivir monophosphate, which covalently binds at the 3′ end of the nascent chain. Covalent binding, in a delayed chain termination manner, nonspecifically inhibits the extension of the nascent chain (Yin et al., 2020; Gordon et al., 2020b). Inhibitory effects on delayed chain termination have also been observed in EBOV or MERS-CoV infection (Tchesnokov et al., 2019; Gordon et al., 2020a). Further investigations revealed that this inhibitory mechanism aims to prevent cleavage by viral nucleic acid exonuclease (Agostini et al., 2018). However, due to controversies in clinical trials, remdesivir has yet to be widely used (Lu et al., 2021). Furthermore, some drugs used to treat hepatitis, such as ribavirin, galidesivir, and sofosbuvir, have been shown in laboratory studies to inhibit the activities of RdRps from other RNA viruses (Maag et al., 2001; Kirby et al., 2015; Elfiky, 2020; Picarazzi et al., 2020; Deshpande et al., 2023). However, the current drugs repurposing for the treatment of other viral diseases requires further investigation.

Favipiravir and molnupiravir are two antiviral drugs developed for influenza that exert inhibitory effects on viral replication. They have also exhibited some degree of inhibitory effects on SARS-CoV-2 infection (Kabinger et al., 2021; Peng et al., 2021), which has led to widespread attention and emergency FDA authorization during the COVID-19 pandemic (Ashwlayan et al., 2022). However, the subsequent in-depth studies on these two drugs revealed that favipiravir requires more clinical samples to evaluate treatment timing, dosage, and side effects, whereas, molnupiravir is likely to induce a high proportion of G-to-A nucleotide mutations in SARS-CoV-2, potentially allowing mutated strains to continue spreading after treatment (Sanderson et al., 2023; Korula et al., 2024). Therefore, it is necessary to consider new avenues for antiviral drugs from multiple perspectives. Recently, nanobody based on the crystal structure of dimerization RdRp from IAV, which targets the dimerization interface, have been developed; the nanobody disrupts the normal conformation of the dimer and affects the synthesis of IAV vRNA, thereby inhibiting viral replication (Fan et al., 2019). The antiviral effects of nanobodies against porcine reproductive and respiratory syndrome virus (PRRSV), Chikungunya virus (CHIKV), and HCV, which target key viral replication proteins, have also been demonstrated (Thueng-in et al., 2012; Wang et al., 2019; Deng et al., 2022). The innovative treatment approach brings new hope to the field of antiviral drugs. Natural nanobodies offer numerous advantages, including a compact structure, high specificity, and good biocompatibility, serving as an ideal choice for treating viral diseases. Therefore, the application of natural nanobodies in the treatment of viral diseases undoubtedly represents a promising direction for future exploration and development.

## 5 Conclusions and prospects

Viral replication is a crucial stage in the virus life cycle and typically involves multiple steps, encompassing the mechanisms of viral genome replicases, interactions with cellular proteins and the coordinated orchestration of various biochemical reactions. Reportedly, various DNA or RNA viruses can form unique ROs within living cells by hijacking specialized subcellular structures of host cells, containing both virus-encoded replicases and cellular proteins (Wu et al., 2022). Research on viral replicases has gradually become an important approach for understanding and intervening in viral infections. Elucidation of the structural and functional aspects of viral replicases provides insights into the virus life cycle.

Recently, although the structures and functions of viral proteins involved in viral replication have been successfully dissected and extended to the research on the polymerase holoenzyme of MPXV, the replication-transcription core enzyme of SARS-CoV-2, the replication initiation and elongation complexes of DENV, and the critical aa involved in the assembly of the HCV replication enzyme complex (Peng et al., 2020, 2023a; Zhang et al., 2021b; Osawa et al., 2023), there are still several unknowns on the composition of replication complexes in various viruses. Further investigation into the spatial configuration of viral replication complexes and the modes of interaction among replicases could elucidate the molecular events of viral replication. Additionally, viral replication is a dynamic process, among which are the assembly and disassembly of replication complexes. Therefore, the application of new technologies, such as single-molecule imaging, single-particle tracking, and time-resolved cryo-electron microscopy, is necessary to track the dynamic assembly and disassembly of replication complexes and to study how cellular environments and viral factors regulate these processes, a potential hotspot for future study on viral replication.

For some highly mutable RNA viruses, traditional antiviral drugs often face efficacy limitations. The mutability of these viruses makes them prone to developing resistance, resulting in poor treatment outcomes. Through in-depth research on viral replicases, novel conserved antiviral drug targets can be discovered, leading to the design of more effective drugs against viral infections. Recently, numerous antiviral drugs targeting SARS-CoV-2 have been reported, many of which inhibit the activity of the viral RdRp (Elfiky, 2020; Gordon et al., 2020b; Kabinger et al., 2021; Peng et al., 2021), thereby hindering viral RNA synthesis. These RdRp-targeting drugs for COVID-19 have been urgently utilized during the pandemic and have shown certain therapeutic effects. However, antiviral drugs targeting viral replicases do not always work well. For instance, the antiviral drug ACV, which targets the herpesviral DNA polymerase, has encountered issues, such as decreased drug efficacy after 40 years of use (Majewska and Mlynarczyk-Bonikowska, 2022), indicating that changes in factors, such as the host’s internal environment, can lead to corresponding alterations in viral replicases. Therefore, it is necessary to explore other conserved active regions of the herpesviral DNA polymerase or find new viral replicase targets. The viral DNA sliding clamp protein, a critical component of the replication machinery, however, there is currently a lack of the drugs targeting DNA sliding clamp, which represents a promising approach for the development of future antiviral drugs against DNA viruses. Additionally, with the continuous development of biotechnology, gene editing, protein engineering, and novel delivery materials can be utilized in the future to design relevant drugs targeting key replicases in viral replication complexes, thereby combating the viral replication.

In summary, increasing research on viral replicases and replication complexes is crucial for addressing current highly pathogenic viruses and potential future ‘X’ viruses. The research not only aids in elucidating viral replication mechanisms but also provides novel therapeutic approaches for viral diseases, thereby exerting profound impacts on global public health and medical fields.

## Author contributions

HD: Conceptualization, Data curation, Formal analysis, Funding acquisition, Investigation, Methodology, Project administration, Resources, Software, Supervision, Validation, Visualization, Writing – original draft, Writing – review & editing. HC: Conceptualization, Data curation, Formal analysis, Funding acquisition, Investigation, Methodology, Project administration, Resources, Software, Supervision, Validation, Visualization, Writing – original draft, Writing – review & editing. YW: Writing – review & editing. JL: Writing – review & editing. JD: Writing – review & editing. L-FL: Writing – review & editing. H-JQ: Conceptualization, Data curation, Formal analysis, Funding acquisition, Investigation, Methodology, Project administration, Resources, Software, Supervision, Validation, Visualization, Writing – original draft, Writing – review & editing. SL: Conceptualization, Data curation, Formal analysis, Funding acquisition, Investigation, Methodology, Project administration, Resources, Software, Supervision, Validation, Visualization, Writing – original draft, Writing – review & editing.
